# Prospective Comparison Between Shotgun Metagenomics and Sanger Sequencing of the 16S rRNA Gene for the Etiological Diagnosis of Infections

**DOI:** 10.3389/fmicb.2022.761873

**Published:** 2022-04-06

**Authors:** Claudie Lamoureux, Laure Surgers, Vincent Fihman, Guillaume Gricourt, Vanessa Demontant, Elisabeth Trawinski, Melissa N’Debi, Camille Gomart, Guilhem Royer, Nathalie Launay, Jeanne-Marie Le Glaunec, Charlotte Wemmert, Giulia La Martire, Geoffrey Rossi, Raphaël Lepeule, Jean-Michel Pawlotsky, Christophe Rodriguez, Paul-Louis Woerther

**Affiliations:** ^1^Microbiology Unit, Department of Diagnostic, Prevention and Treatment of Infections, Henri Mondor Hospital, AP-HP, University of Paris-Est-Créteil, Créteil, France; ^2^Department of Bacteriology, Virology, Hospital Hygiene, and Parasitology-Mycology, Brest University Hospital, Brest, France; ^3^Univ Brest, INSERM, EFS, UMR 1078, GGB, Brest, France; ^4^GHU AP-HP Sorbonne Université, Service des Maladies Infectieuses et Tropicales, Hôpital Saint-Antoine, Paris, France; ^5^INSERM U955, IMRB Institute, University of Paris-Est Créteil, Créteil, France; ^6^EA 7380 Dynamyc, EnvA, UPEC, University of Paris-Est Créteil, Créteil, France; ^7^NGS Platform, Henri Mondor Hospital, AP-HP, and IMRB Institute, University of Paris-Est-Créteil, Créteil, France; ^8^Antimicrobial Stewardship Unit, Diagnostic, Prevention and Treatment of Infections Department, Henri Mondor Hospital, AP-HP, University of Paris-Est-Créteil, Créteil, France

**Keywords:** shotgun metagenomics, molecular diagnostic, pathogen identification, Sanger sequencing of the 16S rRNA gene, microbial documentation

## Abstract

Bacteriological diagnosis is traditionally based on culture. However, this method may be limited by the difficulty of cultivating certain species or by prior exposure to antibiotics, which justifies the resort to molecular methods, such as Sanger sequencing of the 16S rRNA gene (Sanger 16S). Recently, shotgun metagenomics (SMg) has emerged as a powerful tool to identify a wide range of pathogenic microorganisms in numerous clinical contexts. In this study, we compared the performance of SMg to Sanger 16S for bacterial detection and identification. All patients’ samples for which Sanger 16S was requested between November 2019 and April 2020 in our institution were prospectively included. The corresponding samples were tested with a commercial 16S semi-automated method and a semi-quantitative pan-microorganism DNA- and RNA-based SMg method. Sixty-seven samples from 64 patients were analyzed. Overall, SMg was able to identify a bacterial etiology in 46.3% of cases (31/67) vs. 38.8% (26/67) with Sanger 16S. This difference reached significance when only the results obtained at the species level were compared (28/67 vs. 13/67). This study provides one of the first evidence of a significantly better performance of SMg than Sanger 16S for bacterial detection at the species level in patients with infectious diseases for whom culture-based methods have failed. This technology has the potential to replace Sanger 16S in routine practice for infectious disease diagnosis.

## Introduction

Molecular biology-based approaches, including next-generation sequencing (NGS), have changed the face of biological diagnosis in many medical areas (Couto et al., 2018; Simner et al., 2018). However, culture-based methods are still considered as the “gold standard” in Bacteriology, not only to identify pathogenic microorganisms, but also to determine their susceptibility to antimicrobial agents. In case of prior administration of antimicrobial drugs or when fastidious microorganisms are involved, culture-based approaches can yield falsely negative results. In that case, DNA or RNA detection, which could be less impacted, would be more appropriate. Amplification of the universal bacterial 16S rRNA gene and Sanger sequencing of the PCR products (Sanger 16S) is by far the most widely used molecular method for bacterial detection in clinical microbiology laboratories. However, the Sanger 16S approach has biases and limitations (Church et al., 2020). First this method is poorly adapted to detect more than one bacterial species per primers pair, which may be limiting in case of polymicrobial infections (Kommedal et al., 2009). Second, like for any targeted methods, the choice of the primers used for the PCR reaction can significantly impact the sensitivity and specificity of the amplification step. Third, discrimination at the species level is difficult for certain bacterial genera, according to the length of the DNA amplicon (e.g., *Staphylococci, Enterococci*) (Church et al., 2020), despite the fact that this information is essential for infectious gateway documentation and adequate antibiotic treatment prescription. Last, the prediction of antibiotic resistance is impossible (Abayasekara et al., 2017; Schriefer et al., 2018). These limitations make it necessary to evaluate alternative methods. Among them, NGS-based approaches, as shotgun metagenomics (SMg) or amplification of the universal bacterial 16S rRNA gene and NGS sequencing of the PCR products (NGS 16S), appear to be particularly promising. Focusing on SMg, recent studies have demonstrated its utility for the etiological diagnosis of infections (Miao et al., 2018; Filkins et al., 2020; Gu et al., 2021; Miller and Chiu, 2021), including meningitidis/encephalitis (Wilson et al., 2019; Rodino et al., 2020), necrotizing soft-tissue infections (Rodriguez et al., 2020c), pneumonia (Hilton et al., 2016; Zhou et al., 2021), bloodstream infections (Grumaz et al., 2016), bone and joint infections (Street et al., 2017; Thoendel et al., 2018; Gamie et al., 2021; Goswami et al., 2021) or fever with unknown origin (Fu et al., 2022). Moreover, because SMg has the capacity to generate the full-length sequence of bacterial genomes (unlike 16S approaches), this method could be used for the study of antimicrobial susceptibility and molecular typing. For all these reasons, Sanger 16S could be replaced by SMg, with the expectation that cost will continue to decrease with the advent of new technological solutions (Forbes et al., 2017).

The aim of this work was to compare the performance of SMg and Sanger 16S for bacterial detection in routine practice. We conducted a prospective study comparing two molecular methods for bacterial identification: a broadly used semi-automated targeted approach based on amplification of the 16S rRNA gene and Sanger sequencing of the PCR products, and a semi-quantitative pan-microorganism DNA- and RNA-based SMg method.

## Materials and Methods

### Study Population and Samples

All patients’ samples, negative in culture at the time of inclusion, for which Sanger 16S analysis was requested and sent to our laboratory between November 1, 2019 and April 30, 2020, were included in this prospective, comparative, observational and non-inferiority study. Culture negativity of the sample was verified at the time of inclusion. The following sociodemographic and clinical parameters were recorded: age, sex, and antibiotic administration before or at the time of sample processing. The samples were prospectively processed with Sanger 16S and SMg approaches, both of which are available for routine care. The final diagnosis of infection was made by clinicians according to the clinical and biological arguments.

This study protocol was approved by the research ethics committee of the Henri Mondor University Hospital (registration number 00011558). According to French law, complete information about the study was given to each patient. This observational study was conducted under the “Méthodologie de Référence 004” (registration number 3312300420) and was declared to the “Commission Nationale de l’Informatique et des Libertés.” The Sanger 16S approach was performed first on the samples. Samples were stored at +4°C before Sanger 16S then stored at −20°C before SMg.

### Amplification of the 16S rRNA Gene and Sanger Sequencing of the PCR Products

The semi-automated method UMD-SelectNA CE-IVD kit (Molzym GmbH, Germany) was used for the 16S approach, according to the manufacturer’s recommendations. Briefly, 1 mL of sample was pre-treated with protease and a chaotropic buffer lysing human cells, then subjected to DNase to degrade human nucleic acids. Bacterial DNA was subsequently extracted after a proteinase K treatment by means of a magnetic beads-driven procedure on the Arrow instrument (Nordiag, Oslo, Norway). A control (DNA fragment, sequence not provided by the manufacturer) was added to all samples during DNA extraction to detect the presence of potential PCR inhibitors. Extracts were stored at +4°C until real-time PCR analysis (storage at −20°C if real-time PCR not performed immediately after extraction). PCR amplifications targeting the hypervariable region V3–V4 of the bacterial 16S rRNA gene (primer’s sequences not provided by the manufacturer) and the control (primers’ sequences not provided by the manufacturer) were performed for each sample. The reaction mix comprised 8.0 μL of primers targeting 16S rRNA gene (2.5x concentrated solution), 2.0 μL of DNA staining solution, 6.0 μL of free DNA water, 0.8 μL of Taq DNA polymerase (MolTaq 16S, Molzym GmbH, Germany). A final reaction volume of 20 μL (16 μL of mastermix and 4 μL of DNA extract) was used for the PCR reaction. Real-time PCR was performed with a LightCycler 480 instrument (Roche, Basel, Switzerland) under the following conditions: 95°C for 1 min, 40 cycles at 95°C for 5 s, 55°C for 5 s, and 72°C for 25 s, followed by a melting curve analysis (65–95°C). Samples with a melting temperature value between 86°C(and 90°C were considered positive. PCR products from the positive samples were purified with the MinElute PCR purification kit (Qiagen, Hilden, Germany). Sequencing primers included in the UMD-SelectNA CE-IVD kit (one primer pair for Gram-positive and one for Gram-negative; primer’s sequences not provided by the manufacturer) were used for Sanger sequencing on an ABI 3130xl instrument (Thermo Fisher Scientific, Waltham, United States). Sequences were initially aligned to the SepsiTest BLAST database^1^ and subsequently, if no bacterial species were identified, to the Quick BioInformatic Phylogeny of Prokaryotes database.^2^ Sequences with ≥ 97–99% or ≥ 99% identity to the database were assigned to the genus or species level, respectively, according to the manufacturer’s instructions (Wellinghausen et al., 2009). In certain cases, when the database assigned more than one species belonging to the same species group with ≥ 99% identity, the bacterium was identified at the group level. When assignation was < 97%, no result was provided.

### Shotgun Metagenomics

The SMg strategy used in this study consists of an in-house semi-quantitative pan-microorganism DNA- and RNA-based method, called MetaMIC, that has been previously described (Rodriguez et al., 2020c). This approach has received ISO 15189 certification and is routinely used in our laboratory for the etiological diagnosis of complex infectious syndromes (Rodriguez et al., 2020a,b; Courbin et al., 2022). Briefly, 400 μL of samples were pre-treated with a combination of chemical cell disruption and bead homogenization. Nucleic acids were then extracted using the automated QIASymphony instrument with the DSP DNA Mini kit (Qiagen, Hilden, Germany). Extracts were stored at +4°C until libraries preparation (storage at −20°C if libraries not performed immediately after extraction). DNA libraries were prepared using 5 μL extract at 0.2 ng/μL and Nextera XT DNA kit (Illumina, San Diego, United States). RNA libraries were prepared in parallel using 10 μL extract at 10 ng/μL and RNA Human RiboZero TruSeq Stranded Total RNA Library Prep Kit (Illumina, San Diego, United States). The quality and quantity of each library were evaluated by means of a D1000 ScreenTape on a TapeStation (Agilent, Santa Clara, United States) and the Quant-it dsDNA Assay kit on Varioskan LUX instrument (Thermo Fisher Scientific, Waltham, United States), respectively. Then, DNA and RNA libraries were normalized to equal concentrations (final concentration: 2.1 pM) before pooling, denaturation and paired-end sequencing using the NextSeq 500/550 High Output Kit v2.5, 2 × 150 bp on a NextSeq500 instrument (Illumina, San Diego, United States). An environmental control (molecular grade water) and a positive control including Gram-positive and -negative bacteria and fungi (ZymoBIOMICS Microbial Community Standards D6300, Zymo Research, Irvine, United States) were included in each SMg run and processed through the entire protocol to evaluate the performance of SMg. A minimal number of reads (10 million) was required for DNA or RNA libraries, with the exception of low cell matrices (i.e., cerebrospinal fluid,…). After sequencing, fastq files from both libraries (DNA and RNA sequence data) were analyzed by means of our in-house and patented MetaMIC software (V2.2.1), composed of a mosaic of modules.^3^ The patent has been registered at European Patent Office at PCT application number: PCT/FR2020/052193 (Rodriguez et al., 2020d). All reads with Phred scores lower than 20 for at least one nucleotide were removed and a Phred score of 30 (Q30) had to be reached for at least 75% of reads for one sample according to the manufacturer’s recommendations. Human reads were removed using the hg19 database. Identification of non-human reads was performed by means of a database dedicated to MetaMIC (derived from the NCBI nt database, including bacterial, viral and fungal reads but cleaned of mammals, insects, plants, and synthetic plasmids reads). All reads were grouped by taxonomic number and their genetic distances from each other were checked to rule out aberrant reads. A run was considered valid if each microorganism of the positive control was detected. For each sample, DNA libraries were first used for bacterial detection. A sequence background noise was evaluated by calculating a limit of “blanck” and a limit of detection (LOD), based on the results of the environmental control. Each identification whose count had a value greater than the LOD was compared to the count of the reads obtained in the environmental control. Only the identifications superior to the LOD and the environmental control were kept as positive. Results were considered positive if one (or more) bacterium compatible with the suspected infectious process was identified from sterile anatomical sites (i.e., bone and joint, cardiovascular or central nervous system samples). In case of samples from sites normally colonized by commensal bacteria (i.e., pulmonary, intra-abdominal, genito-urinary, and skin and soft tissue samples), only classical pathogens were reported. Strictly pathogenic bacteria (e.g., *Mycobacterium tuberculosis*) were considered regardless of the number of DNA reads. Possible pathogenic bacteria with a low bacterial load (e.g., *Staphylococcus aureus*) were considered if corresponding reads were also found in RNA libraries (Supplementary Table 1). In a second time, results were interpreted by Infectious Disease specialists as “probable sample contaminant” or “pathogen,” based on international recommendations and the combination of clinical, radiological, biological, and therapeutic evidences. In case of positive identification, semi-quantification was determined using the ratio between bacterial and human reads. Bacterial reads are publicly available on GenBank (SAMN16686554–SAMN16686620).

### Statistical Analysis

The Mc Nemar test was used to compare the Sanger 16S and SMg methods with an alpha risk considered significant when less than 0.05. As both methods are not considered as molecular “gold standard” for bacterial identification, sensitivities of the Sanger 16S and SMg approaches were evaluated by comparing the results provided by one single method with those obtained by the sum of the information generated by both methods.

## Results

### Patient and Sample Characteristics

**FIGURE 1:**
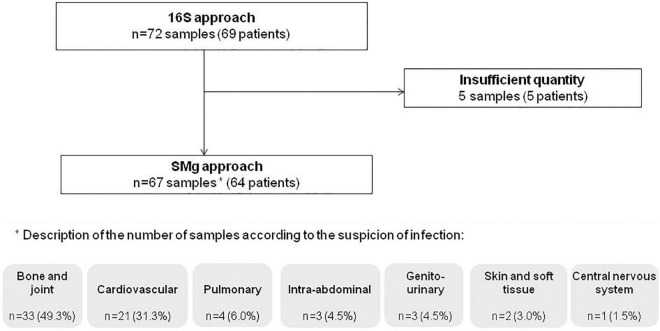
Flow chart of the patients and samples included in the study.

After exclusion of five samples due to a lack of material, 67 samples from 64 patients were included in the study. The study population was composed of 57.8% of males (37/64) and the median age was 62.5 years (range: 19–96). Indications for Sanger 16S prescription were suspicion of bone and joint infection (49.3%; 33/67), cardiovascular infection (31.3%; 21/67), pulmonary infection (6.0%; 4/67), intra-abdominal infection (4.5%; 3/67), genito-urinary infection (4.5%; 3/67), skin and soft tissue infection (3.0%; 2/67), and central nervous system infection (1.5%; 1/67) (Figure 1). Previous exposure to antibiotics (1 month before and/or at the time of sampling) was reported in 62.5% of patients (40/59) [data not available for 7.8% of them (5/64)].

### Amplification of the 16S rRNA Gene and Sanger Sequencing of the PCR Products

**FIGURE 2:**
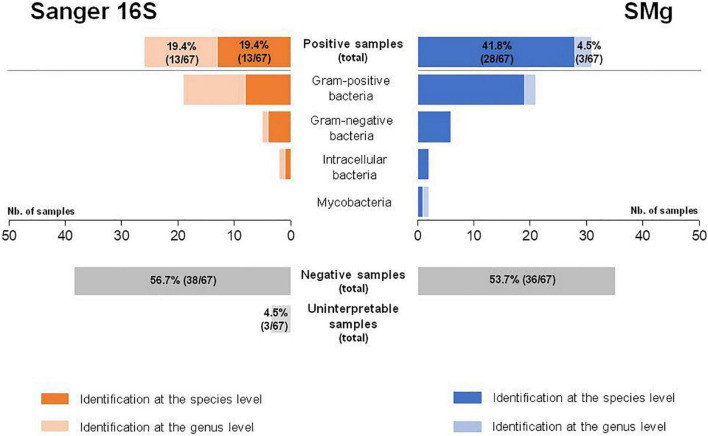
Comparison of Sanger sequencing of the 16S rRNA gene (Sanger 16S) and shotgun metagenomics (SMg) for their ability to identify bacteria at the genus and species levels.

A total of 38.8% of samples (26/67) were positive by Sanger 16S, of which 50.0% (13/26) were identified at the genus level and 50.0% (13/26) at the species level (Table 1). Among these 26 bacteria identified, 73.1% (19/26) were Gram-positive, 19.2% (5/26) were Gram-negative and 7.7% (2/26) were intracellular (Figure 2). No mycobacteria were found. The Sanger 16S approach was uninterpretable for three samples (control failure because of PCR inhibitors, despite dilution of the extracted DNA) (Supplementary Table 1).

**TABLE 1:** Clinical and biological data of the 34 positive samples detected positive with Sanger sequencing of the 16S rRNA gene (Sanger 16S) and/or shotgun metagenomics (SMg).

Suspected type of infection (%;n/N)*	Sample type	Antibiotic therapy**	Identification by SMg	Identification by Sanger 16S	Clinical diagnosis
Bone and joint (48.5%; 16/33)	Joint fluid	N	*Klebsiella pneumoniae*	Negative	Septic arthritis
	Joint fluid (Knee)	Y	*Mycoplasma hominis*	*Mycoplasma hominis*	Septic arthritis
	Joint fluid (Knee)	N	*Neisseria meningitidis*	*Neisseria meningitidis*	Septic arthritis
	Joint fluid	N	*Staphylococcus aureus*	*Staphylococcus aureus*	Septic arthritis
	Joint fluid	Y	*Streptococcus dysgalactiae*	*Streptococcus dysgalactiae*	Septic arthritis
	Joint fluid (Knee)	Y	Negative	*Streptococcus* sp.**^C^** *Moraxella* sp.**^C^**	Microcrystalline arthritis
	Biopsy (Tissue: sternum)	N	*Corynebacterium* sp.**^C^**	Negative	Non-documented infection
	Biopsy (Tissue: hip)	N	*Cutibacterium acnes*	Negative	Prosthetic joint infection
	Biopsy (Tissue: hip)	Y	*Escherichia coli*	*Escherichia coli*	Prosthetic joint infection
	Biopsy (Tissue: elbow)	Y	*Mycobacterium tuberculosis*	Negative	Osteoarthritis
	Biopsy (Tissue: foot)	Y	*Staphylococcus aureus*	*Staphylococcus aureus*	Osteoarthritis
	Biopsy (Tissue: spine)	N	*Staphylococcus aureus*	*Staphylococcus aureus*	Prosthetic joint infection
	Biopsy (Tissue)	Y	Negative	*Streptococcus* sp.	Septic arthritis
	Abscess	N	*Cutibacterium acnes*	*Cutibacterium acnes*	Prosthetic joint infection
	Abscess (Knee)	Y	Negative	*Pseudomonas fluorescens* group**^C^**	Non-documented septic arthritis
	Abscess (Psoas)	Y	*Staphylococcus aureus*	*Staphylococcus aureus*	Vertebral osteomyelitis
Cardiovascular (61.9%; 13/21)	Biopsy (Tissue: aortic aneuvrysm)	Y	*Bacteroides fragilis*	*Bacteroides fragilis*	Endocarditis
	Biopsy (Tissue: vegetation)	N	*Bartonella quintana*	*Bartonella* sp.	Endocarditis
	Biopsy (Tissue: vegetation)	Y	*Enterococcus faecalis*	*Enterococcus* sp.	Endocarditis
	Biopsy (Tissue: vegetation)	Y	*Staphylococcus aureus*	*Staphylococcus* sp.	Endocarditis
	Biopsy (Tissue: aortic valve)	Y	*Staphylococcus epidermidis*	Negative	Endocarditis
	Biopsy (Tissue: valve prosthesis)	N	*Streptococcus anginosus*	*Streptococcus milleri* group	Endocarditis
	Biopsy (Tissue: vegetation)	Y	*Streptococcus dysgalactiae*	β-haemolytic *Streptococcus*	Endocarditis
	Biopsy (Tissue: mitral valve)	Y	*Streptococcus gordonii*	*Streptococcus gordonii*	Endocarditis
	Biopsy (Tissue: vegetation)	Y	*Streptococcus mitis* group	*Streptococcus* sp.	Endocarditis
	Biopsy (Tissue: carotid)	Y	*Streptococcus pneumoniae*	α-haemolytic *Streptococcus*	Aortitis
	Biopsy (Tissue)	Y	*Streptococcus pyogenes*	*Streptococcus pyogenes*	Endocarditis
	Biopsy (Tissue: aortic valve)	Y	*Streptococcus sanguinis*	*Streptococcus mitis* group	Endocarditis
	Abscess (Mediastinum)	Y	*Cutibacterium acnes* (associated with cutaneous flora)	Negative	Mediastinitis
Intra-abdominal (100%; 3/3)	Abscess (Liver)	Y	*Fusobacterium nucleatum*	*Fusobacterium nucleatum*	Liver abscess
	Abscess (Liver)	Y	*Klebsiella pneumoniae*	Negative	Liver abscess
	Abscess (Intra-abdominal)	Y	*Staphylococcus lugdunensis*	*Staphylococcus* sp.	Abdominal abscess
Genito-urinary (33.3%; 1/3)	Abscess (Kidney)	Y	*Enterococcus faecalis*	*Enterococcus* sp.	Kidney graft abscess
Skin and soft tissue (50.0%; 1/2)	Granuloma	N	*Mycobacterium tuberculosis* complex	Negative	Post-BCG infection

*Percentage of positive samples for each suspected type of infection in at least one method. **One month before and/or at the time of sampling; N, No; Y, Yes;

*^C^, Probable sample contaminant; SMg, Shotgun metagenomics; Sanger 16S, Amplification of the 16S rRNA gene and Sanger sequencing of the PCR products.*

### Shotgun Metagenomics

Phred score (Q30) for all sample and positive control results for all experiments were in keeping with the specifications of the method for the SMg run validation. The median depth of SMg sequencing was 29,159,912 DNA reads (range: 435,424–159,677,808) and 19,426,688 RNA reads (range: 1,970–49,574,336) per sample. The total number of reads for DNA library was lower than 10 million for three samples (Supplementary Table 1). In total, 46.3% of the samples (31/67) were positive by SMg, of which 9.7% (3/31) were identified at the genus level and 90.3% (28/31) at the species level (Table 1). Among the 31 bacteria detected, 67.6% (21/31) were Gram-positive, 19.4% (6/31) were Gram-negative, 6.5% (2/31) were intracellular, and 6.5% (2/31) were mycobacteria (Figure 2). Semi-quantification by metagenomics provided high bacterial loads (ratio > 10^–2^) for 3.3% (1/31) of cases, intermediate bacterial loads (ratio = 10^–2^–10^–5^) for 41.9% (13/31) of cases, and low bacterial loads (ratio < 10^–5^) for 54.8% (17/31) of cases (Supplementary Table 1). No viral or fungal DNA or RNA reads were detected in any of the samples.

### Comparison of Sanger Sequencing of the 16S rRNA Gene vs. Shotgun Metagenomics

Bacterial identification was consistent in all the 23 samples found positive with both methods. However, 39.1% of them (9/23) could be identified at the species level by SMg, whereas Sanger 16S only provided the genus name in all of them. SMg detected bacteria in 88.5% (23/26) of Sanger 16S-positive samples. For the remaining three samples for which SMg was negative, the four bacteria not detected were: *Streptococcus* sp. from a biopsy; a mix of *Streptococcus* sp. and *Moraxella* sp. from a joint fluid and *Pseudomonas fluorescens* group from an abscess (Table 1). Except for *Streptococcus* sp., the others bacteria were considered as possible contaminants (human and environmental contaminants, respectively) and were not taken into account for patient care. Conversely, SMg identified eight bacteria in 19.5% (8/41) of samples negative with the Sanger 16S approach. Among these bacteria, 25.0% (2/8) were identified at the genus level and 75.0% (6/8) at the species level (Figure 2). Seven of these bacteria not detected by Sanger 16S were considered relevant and taken into account for patient care.

Overall, SMg was able to identify a bacterial etiology in 31/67 samples vs. 26/67 with Sanger 16S (*p* = 0.25, Mc Nemar Test). Interestingly, this difference reached significance when only the results obtained at the species level were compared (28/67 vs. 13/67; *p <* 0.001, Mc Nemar Test) (Figure 2). Sensitivities for the identification at the genus and at the species level, respectively, were 91.2% (31/34) and 82.4% (28/34) for SMg and 76.5% (26/34) and 38.2% (13/34) for Sanger 16S. Considering only the clinically relevant results (i.e., exclusion of bacteria considered as probable sample contaminant), the performance for the identification at the genus and at the species level, respectively, were 96.8% (30/31) and 90.3% (28/31) for SMg and 77.4% (24/31) and 41.9% (13/31) for Sanger 16S.

## Discussion

The high performance (sensitivity and specificity) of SMg has been recently highlighted for the etiological diagnosis of a number of infectious diseases (Miao et al., 2018; Filkins et al., 2020; Gu et al., 2021; Miller and Chiu, 2021), including bone and joint infections (Street et al., 2017; Thoendel et al., 2018; Gamie et al., 2021; Goswami et al., 2021), meningitis and encephalitis (Wilson et al., 2019; Rodino et al., 2020), or necrotizing fasciitis (Rodriguez et al., 2020c). Indeed, by allowing the detection and quantification of an unlimited panel of microorganisms, including bacteria, viruses, fungi, or parasites without *a priori* orientation, SMg is capable to identify the etiology of any infectious disease, including those due to microorganisms that have never been previously described in human infections (Rodriguez et al., 2020b). However, SMg has been barely implemented in the routine practice of microbiology laboratories, because of its cost and the specialized skills required for bioinformatics, biological and clinical interpretation of sequencing results. Nowadays, Sanger 16S remains the most widely used molecular tool to detect and identify bacteria from clinical samples when culture-based methods have failed. Nevertheless, the intrinsic performance of Sanger 16S and SMg in detecting bacteria and identifying them at the species level have rarely been assessed. A further comparative study between both approaches (five patients) was in favor of the superiority of SMg for bacteriological diagnosis (Gu et al., 2021).

In the present study, SMg detected bacteria in 19.5% of samples (8/41) that were found negative by Sanger 16S. Among these samples, the bacterial identification allowed a documentation in line with the clinical presentation in seven cases: *Cutibacterium acnes* in a case of a prosthetic joint infection; *Cutibacterium acnes* in a case of nosocomial mediastinitis; *Klebsiella pneumoniae* in a case of septic arthritis and liver abscess, respectively; *Mycobacterium tuberculosis* in a case of osteoarthritis; *Mycobacterium tuberculosis* complex in a case of post-BCG infection and *Staphylococcus epidermidis* in a case of endocarditis (Table 1). Conversely, *Corynebacterium* sp., identified in one sample (tissular biopsy) taken for suspected bone infection, was considered as a sample contaminant by cutaneous flora in absence of clinical argument of infection due to this bacterium. Importantly, two samples that were negative by Sanger 16S were found positive for the presence of mycobacteria by SMg. This result, in keeping with the ability of SMg to detect mycobacteria (Miao et al., 2018; Zou et al., 2022) whereas 16S may lack specificity (Patel et al., 2000), highlights a key input of the SMg approach, in particular in situations where mycobacteriosis was not initially suspected from the clinical presentation.

Four bacteria (*Streptococcus* sp., *Streptococcus* sp. associated with *Moraxella* sp., and *Pseudomonas fluorescens* group) were detected by Sanger 16S, but not by SMg, and identified only at the genus level. Whereas one was considered as pathogenic (*Streptococcus* sp. in a patient with septic arthritis), *Pseudomonas fluorescens* group and the mix of *Streptococcus* sp. and *Moraxella* sp. were considered as probable environmental and human contaminants, respectively (contamination at the time of the sampling or during sample handling in the laboratory).

In all the 23 samples found positive with both methods, bacterial identification was consistent but SMg was more efficient than Sanger 16S to provide identification at the species level in nine samples (39.1%) identified only at the genus level by Sanger 16S. In comparison to the V3–V4 region of the 16S rRNA gene, the information provided by SMg allows covering a broader portion of the bacterial genome, including additional genes which may provide critical information to determine the species with precision. Accordingly, certain species are particularly difficult to identify by 16S rRNA gene sequencing (e.g., some species of the genus *Streptococcus*; Picard et al., 2004), compared with SMg approaches that theoretically allows to cover the entire bacterial genome. The ability of SMg to identify bacteria at the species level is of major importance for clinical management, including the choice of antimicrobial molecules and the treatment duration, although this has not been evaluated in this work. This was the case when *Staphylococcus aureus* was identified instead of *Staphylococcus* sp. or when *Enterococcus faecalis* was identified instead of *Enterococcus* sp. in two cardiac valves from patients with infectious endocarditis.

SMg is able to provide much more information than targeted approaches by detecting all potential bacterial and non-bacterial pathogens (virus, fungi) in one test (Wilson et al., 2019; Rodriguez et al., 2020b). As SMg was compared to Sanger 16S in samples from patients with suspected bacterial infections, only bacteria were detected in this work. Although not retrieved in the present study due to the depth of sequencing (minimal number of 10 million of reads per library), information on strain characterization, detection of virulence genes or resistance profile analysis can be theoretically obtained from SMg data (Andersen et al., 2016; Graf et al., 2016; Schmidt et al., 2017).

Considering the technical specifications of both approaches, the timeline has to be taken into account. Currently, in our institution, we estimate that the delay from the start of the analysis to the results is estimated at 2.5 days for Sanger 16S and 5 days for SMg, respectively. Moreover, it should be noted that SMg requires specific skills and expensive equipment; a summary description of the mains steps of the SMg workflow has been recently published (d’Humières et al., 2021). Finally, the cost of both methods is a considerable factor in the decision to realize novel diagnostic approaches in routine practice. Current SMg technology costs about three times more than the Sanger 16S approach in our laboratory with a large NGS platform (estimated SMg price: 200–300 euro per sample, excluding labor). However, these estimations deeply depend on the rate of flow and the size of the platform, the material and the technology used, as well as many other parameters including the country or the period during which the data were processed. Furthermore, it should be noted that most of the cost of SMg (approximately 2/3) is covered by the sequencing step, which is constantly decreasing.

Our study has some limitations. First, the potential bias due to samples freezing-thawing could not be accounted for in the interpretation of the results obtained in this study. Second, in order to make our study useful for most microbiologists and clinicians, we used the semi-automated kit UMD-SelectNA CE-IVD to perform the comparison between Sanger 16S and an accredited SMg approach (ISO 15189), as laboratories need to work with technologies approved by the FDA and/or CE-IVD. However, others methods used in microbiology laboratories for Sanger 16S (“in-house” or targeting other hypervariable regions of the 16S rRNA gene than V3–V4), may have slightly different performance. Moreover, it is important to mention that alternatives to Sanger sequencing using NGS technologies for 16S approach are numerous as amplicon sequencing or targeted capture technologies (Boers et al., 2019; Rassoulian Barrett et al., 2020), but still rarely used in routine practice currently. NGS 16S approaches enable bacterial identification in case of polymicrobial infections (Culbreath et al., 2019). Third, we used the threshold of 97% recommended by the manufacturer for the assignation at the genus level with Sanger 16S (Wellinghausen et al., 2009). However it should be noted that lower thresholds have been reported in others publications (Bemer et al., 2014; Yarza et al., 2014). Fourth, the minimal number of 10 million reads for DNA library was not obtained for three samples (Supplementary Table 1), which may lead to a decrease in the sensitivity of the SMg approach. Fifth, the turnaround time and the impact of our results on clinical care and outcomes were not evaluated. Nevertheless, SMg results were delivered to the clinicians and only the results taken into account in their treatment decisions were considered as true positives, suggesting clinical relevance. Finally, the results delivered to the clinicians depended on the nature of the sample (sterile or possibly colonized sample) and on the bacterium identified (i.e., pathogenicity level). In absence of “gold standard” other than culture, the categorization of the results delivered as true positives depended on the final decision made by the clinicians to consider the result in the diagnosis.

In conclusion, SMg showed a better contribution to bacterial documentation at the species level when compared to Sanger 16S for the etiological diagnosis of infectious syndromes in case of standard cultures negative. These results suggest that SMg may replace in the future Sanger 16S in routine practice to diagnose bacterial infections. Future studies assessing the clinical benefit of SMg and the possible impact on prognosis are, however, still needed.

## Data Availability Statement

The datasets presented in this study can be found in online repositories. The names of the repository/repositories and accession number(s) can be found in the article/Supplementary Material.

## Ethics Statement

The studies involving human participants were reviewed and approved by the Henri Mondor Hospital Institutional Review Board, Créteil, France (approval number 00011558). The patients/participants provided their written informed consent to participate in this study.

## Author Contributions

CL, LS, VF, J-MP, CR, and P-LW contributed in every aspect of this research work including conception, study design, and data analysis and interpretation. VD, ET, NL, and J-ML performed the laboratory experiments. CG and MN’D performed bioinformatics analysis. CL, LS, J-MP, CR, and P-LW wrote the manuscript. LS, CW, GL, GRos, and RL participated in interpretation of data and conflicting results (adjudication committee). VF, CG, and GRoy participated in review of the manuscript. All authors contributed to manuscript revision, read and approved the submitted manuscript.

## Conflict of Interest

CR served as an advisor, and/or speaker for Illumina, and Vela Diagnostics. P-LW has served as speaker for MSD. J-MP served as an advisor, and/or speaker for Abbvie, Gilead, GlaxoSmithKline, Merck, Regulus, and Siemens Healthcare. The remaining authors declare that the research was conducted in the absence of any commercial or financial relationships that could be construed as a potential conflict of interest.

## Publisher’s Note

All claims expressed in this article are solely those of the authors and do not necessarily represent those of their affiliated organizations, or those of the publisher, the editors and the reviewers. Any product that may be evaluated in this article, or claim that may be made by its manufacturer, is not guaranteed or endorsed by the publisher.

## Footnotes

1
www.sepsitest-blast.net

2
https://umr5558-proka.univ-lyon1.fr/lebibi/lebibi-legacy.cgi

3
https://gitlab.com/mndebi/metamic
