# Supplementary material for: Prospective Comparison Between Shotgun Metagenomics and Sanger Sequencing of the 16S rRNA Gene for the Etiological Diagnosis of Infections
Source: Front Microbiol. 2022 Apr 6;13:761873. doi: 10.3389/fmicb.2022.761873 (PMC9020828; doi:10.3389/fmicb.2022.761873)
Supplement: Supplementary file 1 [file Table_1.pdf]

Sample	Sample type	Suspected type of infection	Sanger 16S results	Identification by Sanger 16S	SMg results	Identification by SMg (DNA library)	Number of DNA reads (relative abundance %*)	Bacterial load	Detection of RNA sequences of the bacteria detected by SMg (reads/relative abundance %)	Others bacterial species identified by SMg** (DNA reads/relative abundance %)	Total reads (DNA)	Total human reads (DNA)	Total bacterial reads (DNA)	Total reads (RNA)	Total human reads (RNA)	Total bacterial reads (RNA)
1	Abscess (psoas)	Bone and joint	Positive	*Staphylococcus aureus*	Positive	*Staphylococcus aureus*	53 (88.3)	Low	Yes (30/1.5)	*Klebsiella pneumoniae* (3/5.0)	49,051,690	34,087,606	60	26,863,460	14,038,123	2,068
2	Biopsy (tissue: carotid)	Cardiovascular	Positive	α-haemolytic *Streptococcus*	Positive	*Streptococcus pneumoniae*	47 (58.8)	Low	Yes (2/10)	*Cutibacterium acnes* (14/17.5), *Ralstonia solanacearum* (8/10.0), *Staphylococcus aureus* (5/6.3)	43,878,464	29,729,838	80	116,560	59,119	20
3	Biopsy (tissue: mitral valve)	Cardiovascular	Positive	*Streptococcus gordonii*	Positive	*Streptococcus gordonii*	21,853 (99,2)	Intermediate	Yes (57,626/34.8)	X	37,587,780	24,557,004	22,033	49,574,336	32,562,501	165,797
4	Abscess (liver)	Intra-abdominal	Negative	X	Positive	*Klebsiella pneumoniae*	147 (76.6)	Low	Yes (40/1.7)	*Ralstonia solanacearum (18/9.4), Staphylococcus aureus (9/4.7), Cutibacterium acnes (6/3.1), Streptococcus salivarius (4/2.1)*	24,804,076	11,906,531	192	28,540,204	15,148,646	23,574
5	Biopsy (tissue: elbow)	Bone and joint	Uninterpretable	X	Positive	*Mycobacterium tuberculosis*	10 (2.1)	Low	No	*Cutibacterium acnes (373/79.5), Rhodococcus rhodochrous (12/2.6), Escherichia coli (6/1.3), Pseudomonas putida* group *(6/1.3), Bifidobacterium longum (6/1.3), Propionibacterium* sp *(5/1.1)*	86,974,428	45,885,017	469	1,730	70	0
6	Biopsy (tissue: thrombus)	Cardiovascular	Negative	X	Negative	X	X	X	X	*Cutibacterium acnes (4/80.0)*	30,429,258	17,110,896	5	13,993,974	9,113,432	2,925
7	Joint fluid (knee)	Bone and joint	Negative	X	Negative	X	X	X	X	*Staphylococcus aureus (6/60.0)*	34,015,014	19,155,805	10	15,271,164	7,672,062	29,906
8	Pleural fluid	Pulmonary	Negative	X	Negative	X	X	X	X	*Escherichia coli (11/12.2), Salmonella enterica (10/11.1), Pseudomonas aeruginosa group (10/11.1), Cutibacterium acnes (8/8.9), Staphylococcus aureus (8/8.9), Enterococcus faecalis (6/6.7), Pseudomonas aeruginosa (6/6.7), Bacillus subtilis (4/4.4), Listeria monocytogenes (4/4.4)*	37,090,716	21,023,047	90	22,300,834	12,362,379	20,877
9	Abscess	Bone and joint	Positive	*Cutibacterium acnes*	Positive	*Cutibacterium acnes*	9,498 (99.9)	Intermediate	Yes (3,706/18.6)	X	37,213,620	26,479,436	9,504	7,538,952	5,042,457	19,890
10	Joint fluid (knee)	Bone and joint	Positive	*Neisseria meningitidis*	Positive	*Neisseria meningitidis*	2 (40.0)	Low	Yes (126/1.2)	*X*	11,896,258	7,596,960	5	43,153,296	26,682,092	10,953
11	Biopsy (tissue: mitral valve)	Cardiovascular	Negative	X	Negative	X	X	X	X	*Staphylococcus aureus (6/15.4), Cutibacterium acnes (6/15.4), Pseudomonas aeruginosa group (4/10.3), Acidovorax ebreus (3/7.7)*	40,784,624	27,715,956	39	26,346,200	16,147,978	30,402

12	Biopsy (tissue)	Bone and joint	Positive	*Streptococcus* sp.	Negative	X	X	X	X	*Staphylococcus aureus (4/33.3), Streptococcus sp (4/33.3)*	21,526,680	13,955,159	12	134,554	30,813	18
13	Cerebrospinal fluid	Central nervous system	Negative	X	Negative	X	X	X	X	*Cutibacterium acnes (336/11.2), Thermus scotoductus (303/10.1), Methylobacterium extorquens (208/6.9), Pseudomonas aeruginosa (195/6.5), Delftia tsuruhatensis (165/5.5), Pseudomonas aeruginosa group (136/4.5), Acidovorax sp (105/3.5), Rhodococcus hoagii (96/3.2), Comamonas aquatica (88/2.9), Meiothermus ruber (64/2.1), Cupriavidus basilensis (51/1.7), Acinetobacter junii (49/1.6), Methylobacterium extorquens group (49/1.6), Cupriavidus gilardii (48/1.6), Achromobacter xylosoxidans (46/1.5), Acidovorax ebreus (41/1.4), Stenotrophomonas maltophilia (36/1.2), Comamonas testosteroni (32/1.1), Stenotrophomonas acidaminiphila (31/1.0), Sphingobium hydrophobicum (31/1.0), Methylobacterium zatmanii (30/1.0), Delftia sp (30/1.0)*	16,264,858	10,104,534	2,994	37,012,206	13,069,328	1,582,075
14	Biopsy (tissue: vegetation)	Cardiovascular	Positive	β-haemolytic *Streptococcus*	Positive	*Streptococcus dysgalactiae*	26,147 (99.1)	Intermediate	Yes (2/100)	*X*	25,909,552	16,726,733	26,375	26,624	11,687	2
15	Abscess (sternal)	Cardiovascular	Negative	X	Negative	X	X	X	X	*Cutibacterium acnes (6/24.0), Ralstonia solanacearum (4/16.0), Pseudomonas sp (4/16.0)*	34,214,594	22,950,433	4	24,368	9,150	0
16	Joint fluid	Bone and joint	Negative	X	Negative	X	X	X	X	*Ralstonia solanacearum (6/42.3), Staphylococcus aureus (3/21.4)*	21,124,936	12,243,267	14	31,218,588	19,959,547	19,646
17	Abscess (mediastinum)	Cardiovascular	Negative	X	Positive	Cutaneous flora with *Cutibacterium acnes* predominance	4,903 (83.2)	Intermediate	Yes (1,141/0.05)	*Micrococcus luteus (131/2.2), Staphylococcus aureus (91/1.5), Thermus scotoductus (86/1.5), Pseudomonas aeruginosa (73/1.2)*	2,809,716	1,630,759	5,895	41,448,934	9,880,372	2,129,628
18	Biopsy (tissue: vegetation)	Cardiovascular	Positive	*Streptococcus* sp.	Positive	*Streptococcus mitis* group	351,809 (99.5)	Intermediate	Yes (165,483/94.0)	*X*	36,874,882	24,685,924	353,737	18,897,302	11,929,973	176,022
19	Biopsy (bone)	Bone and joint	Negative	X	Negative	X	X	X	X	*Ralstonia solanacearum (4/22.2)*	20,329,058	13,894,340	18	33,990,062	23,814,801	43,132
20	Abscess (breast)	Skin and soft tissue	Negative	X	Negative	X	X	X	X	*Ralstonia solanacearum (38/46.3), Staphylococcus aureus (24/29.3), Klebsiella aerogenes (12/14.6)*	56,200,522	24,708,571	82	675,606	327,707	10
21	Abscess (knee)	Bone and joint	Negative	X	Negative	X	X	X	X	*Escherichia coli (16/53.3), Ralstonia solanacearum (8/26.7), Staphylococcus aureus (4/13.3)*	24,712,470	9,530,900	30	35,778	15,252	4
22	Pleural fluid	Pulmonary	Negative	X	Negative	X	X	X	X	*Ralstonia solanacearum (48/41.4), Staphylococcus aureus (17/14.7), Cutibacterium acnes (12/10.3), Acinetobacter johnsonii (6/5.2), Sphingobium hydrophobicum (4/3.4)*	159,677,808	79,102,493	116	43,707,740	21,364,169	4,238
23	Biopsy (tissue: vegetation)	Cardiovascular	Positive	*Staphylococcus* sp.	Positive	*Staphylococcus aureus*	50 (90.9)	Low	Yes (2,218/12.2)	X	17,833,068	9,184,346	55	10,796,232	4,386,134	18,160
24	Abscess (kidney)	Genito-urinary	Positive	*Enterococcus* sp.	Positive	*Enterococcus faecalis*	20 (11.6)	Low	Yes (12/0.9)	*Ralstonia solanacearum (67/38.7), Staphylococcus aureus (44/25.4), Uncultured bacterium (6/3.5), Cutibacterium acnes (6/3.5), Acidovorax sp (6/3.5), Enterococcus faecium (6/3.5), Corynebacterium imitans (4/2.3)*	30,262,382	14,285,886	173	41,771,806	19,916,858	1,302

25	Joint fluid	Bone and joint	Positive	*Streptococcus dysgalactiae*	Positive	*Streptococcus dysgalactiae*	36 (43.4)	Low	No	*Ralstonia solanacearum (20/24.1), Staphylococcus aureus (12/14.5), Brevundimonas diminuta 52/2.4), Cupriavidus gilardii (2/2.4), Streptococcus suis (2/2.4), Comamonas aquatica (2/2.4), Actinomyces oris (2/2.4), Streptococcus pyogenes (2/2.4), Cutibacterium acnes (2/2.4)*	27,143,300	17,934,537	83	117,730	67,820	0
26	Joint fluid	Bone and joint	Negative	X	Negative	X	X	X	X	*Ralstonia solanacearum (12/57.1), Staphylococcus aureus (7/33.3)*	45,219,778	30,865,846	21	173,112	55,621	2
27	Pleural fluid	Pulmonary	Negative	X	Negative	X	X	X	X	*Escherichia coli (40/57.1), Ralstonia solanacearum (9/12.9), Pseudomonas aeruginosa group (4/5.7), Thermus scotoductus (3/4.3)*	31,354,508	15,156,842	70	19,069,190	9,106,808	13,783
28	Joint fluid	Bone and joint	Negative	X	Positive	*Klebsiella pneumoniae*	471 (69.7)	Intermediate	No	*Stenotrophomonas maltophilia (85/12.5), Escherichia coli (52/7.7), Stenotrophomonas sp (12/1.8), Salmonella enterica (10/1.5), Klebsiella variicola (7/1.0)*	28,009,902	14,180,503	676	35,458,066	17,404,340	12,443
29	Biopsy (tissue: aortic aneuvrysm)	Cardiovascular	Positive	*Bacteroides fragilis*	Positive	*Bacteroides fragilis*	12,525 (99.4)	Intermediate	Yes (6/75.0)	*X*	48,945,934	32,080,036	12,604	41,732	24,071	8
30	Pleural fluid	Pulmonary	Negative	X	Negative	X	X	X	X	*Cutibacterium acnes (8/40.0)*	18,186,802	8,987,604	20	13,800,400	6,940,325	13,545
31	Abscess	Cardiovascular	Uninterpretable	X	Negative	X	X	X	X	*Cutibacterium acnes (10/34.5), Pseudomonas sp (4/13.8), Ralstonia solanacearum (3/10.3)*	21,444,018	11,630,831	29	398,376	156,628	8
32	Biopsy (tissue: aortic valve)	Cardiovascular	Negative	X	Negative	X	X	X	X	*Ralstonia solanacearum (138/34.5), Staphylococcus aureus (69/17.3), Cutibacterium acnes (42/10.5), Escherichia coli (13/3.3), Cutibacterium granulosum (10/2.5), Acidovorax sp (8/2.0), Campylobacter ureolyticus (8/2.0), Klebsiella pneumoniae (7/1.8), Uncultured bacterium (6/1.5), Staphylococcus epidermidis (6/1.5), Alcaligenes faecalis (5/1.3), Pseudomonas fluorescens (5/1.3), Pseudomonas sp (4/1.0), Moraxella osloensis (4/1.0), Rothia dentocariosa (4/1.0), Diaphorobacter sp (4/1.0), Streptococcus parasanguinis (4/1.0)*	159,086,958	90,146,025	400	32,78	15,383	2
33	Biopsy (tissue: aortic valve)	Cardiovascular	Negative	X	Negative	X	X	X	X	*Cutibacterium granulosum (8/22.2), Cutibacterium acnes (5/13.9), Pseudomonas aeruginosa group (4/11.1)*	28,058,184	13,942,161	36	8,591,480	2,772,464	8,584
34	Biopsy (bone)	Bone and joint	Negative	X	Negative	X	X	X	X	*Ochrobactrum anthropi (12/14.3), Thermus scotoductus (11/13.1), Delftia tsuruhatensis (10/11.9), Cutibacterium acnes (10/11.9), Pseudomonas aeruginosa (8/9.5), Pseudomonas aeruginosa group (6/7.1)*	17,188,902	8,375,224	84	19,426,688	7,669,343	47,127
35	Biopsy (bone)	Bone and joint	Negative	X	Negative	X	X	X	X	*Cutibacterium acnes (30/46.2), Escherichia coli (10/15.4), Anoxybacillus flavithermus (4/6.2), Lactobacillus crispatus (4/6.2)*	21,030,260	11,819,215	65	4,098,466	2,326,183	637

36	Biopsy (tissue: thigh)	Cardiovascular	Negative	X	Negative	X	X	X	X	*Moraxella osloensis* (12/34.3), *Cutibacterium acnes* (6/17.1), *Escherichia coli* (4/11.4), *Ralstonia solanacearum* (3/8.6)	22,452,202	12,827,965	35	1,970	935	2
37	Biopsy (tissue: mastoid)	Bone and joint	Negative	X	Negative	X	X	X	X	*Cutibacterium acnes* (79/65.3), *Staphylococcus hominis* (10/8.3), *Moraxella osloensis* (6/5.0), *Lactobacillus crispatus* (4/3.3), *Kocuria palustris* (4/3.3), *Comamonas aquatica* (2/1.7), *Cutibacterium granulosum* (2/1.7), *Acidovorax ebreus* (2/1.7), *Paracoccus yeei* (2/1.7), *Finegoldia magna* (2/1.7), *Brochothrix thermosphacta* (2/1.7), *Streptococcus suis* (2/1.7), *Rothia dentocariosa* (2/1.7)	30,784,946	20,409,434	121	32,621,686	20,456,556	17,535
38	Biopsy (tissue)	Cardiovascular	Negative	X	Negative	X	X	X	X	*Cutibacterium acnes* (304/60.7), *Staphylococcus hominis* (42/7.0), *Escherichia coli* (29/4.8), *Moraxella osloensis* (25/4.2), *Ralstonia solanacearum* (14/2.3), *Cutibacterium granulosum* (13/2.2), *Staphylococcus epidermidis* (11/1.8), *Streptococcus suis* (7/1.2), *Serratia marcescens* (6/1.0), *Acidovorax sp* (6/1.0), *Methylobacterium extorquens* (6/1.0), *Staphylococcus lugdunensis* (6/1.0), *Staphylococcus aureus* (6/1.0), *Lactobacillus amylophilus* (6/1.0), *Micrococcus luteus* (6/1.0), *Meiothermus silvanus* (6/1.0)	73,133,874	48,786,806	600	15,800,512	9,932,688	16,170
39	Granuloma	Skin and soft tissue	Negative	X	Positive	*Mycobacterium tuberculosis complex*	10 (15.6)	Low	No	*Ralstonia solanacearum* (14/21.9), *Cutibacterium acnes* (6/9.4), *Staphylococcus aureus* (6/9.4), *Escherichia coli* (6/9.4), *Klebsiella pneumoniae* (5/7.8), *Acidovorax sp* (4/6.3), *Capnocytophaga sputigena* (4/6.3)	25,105,672	14,557,253	64	63,048	32,569	0
40	Biopsy (tissue: inguinal)	Genito-urinary	Negative	X	Negative	X	X	X	X	*Escherichia coli* (8/11.9), *Thermus scotoductus* (7/10.4), *Delftia tsuruhatensis* (7/10.4), *Cutibacterium acnes* (6/9.0), *Cupriavidus gilardii* (5/7.5), *Shigella boydii* (4/6.0), *Rhodococcus hoagii* (4/6.0)	24,823,602	15,868,071	67	38,297,470	26,231,331	146,824
41	Joint fluid (knee)	Bone and joint	Positive	*Mycoplasma hominis*	Positive	*Mycoplasma hominis*	24 (70.6)	Low	Yes (161/1.5)	*Cutibacterium acnes* (6/17.6)	34,662,278	16,522,451	34	45,128,954	17,356,452	10,823
42	Biopsy (tissue)	Cardiovascular	Positive	*Streptococcus pyogenes*	Positive	*Streptococcus pyogenes*	102 (94.4)	Low	Yes (326/4.0)	*X*	27,745,268	13,019,717	108	36,336,862	12,847,888	8,187
43	Biopsy (tissue)	Bone and joint	Negative	X	Negative	X	X	X	X	*Kocuria palustris* (569/18.6), *Deinococcus proteolyticus* (442/14.4), *Arsenicicoccus sp* (244/8.0), *Delftia tsuruhatensis* (206/6.7), *Cupriavidus gilardii* (177/5.8), *Cutibacterium acnes* (160/5.2), *Micrococcus luteus* (121/3.9), *Kytococcus sedentarius* (93/3.0), *Achromobacter xylosoxidans* (82/2.8), *Janibacter indicus* (72/2.3), *Acidovorax sp* (49/1.6)	435,424	165,868	3,066	24,382,782	2,642,123	424,889

44	Biopsy (bone)	Bone and joint	Negative	X	Negative	X	X	X	X	*Cutibacterium acnes (66/54.1), Staphylococcus hominis (14/11.5), Corynebacterium singulare (9/7.4), Ralstonia solanacearum (6/4.9), Moraxella osloensis (6/4.9)*	24,264,080	15,685,939	122	21,716,116	13,727,327	17,661
45	Biopsy (tissue: sternum)	Bone and joint	Negative	X	Positive	*Corynebacterium* sp.	200 (53.5)	Low	Yes (845/3.4)	*Cutibacterium acnes (104/27.8), Staphylococcus hominis (14/3.7), Escherichia coli (9/2.4), Lactobacillus crispatus (6/1.6), Staphylococcus epidermidis (5/1.3), Lactobacillus johnsonii (4/1.1), Moraxella osloensis (4/1.1)*	40,880,544	27,563,026	374	13,278,044	8,588,517	24,517
46	Biopsy (tissue: vegetation)	Cardiovascular	Positive	*Enterococcus* sp.	Positive	*Enterococcus faecalis*	509 (82.8)	Low	Yes (524/4.7)	*Cutibacterium acnes (56/9.1), Lactobacillus crispatus (6/1.0)*	30,376,828	19,691,476	615	11,694,134	6,958,777	11,085
47	Biopsy (bone: pubic symphysis)	Bone and joint	Negative	X	Negative	X	X	X	X	*Cutibacterium acnes (274/31.7), Staphylococcus hominis (119/13.8), Moraxella osloensis (52/6.0), Escherichia coli (48/5.5), Microbacterium aurum (46/5.3), Bacillus flexus (46/5.3), Pseudomonas aeruginosa group (18/2.1), Rhizobium sp (18/2.1), Ralstonia solanacearum (16/1.8), Kocuria palustris (16/1.8), Staphylococcus epidermidis (14/1.6), Acinetobacter sp (13/1.5), Lactobacillus crispatus (10/1.2)*	8,925,940	5,549,087	865	28,466,736	19,819,575	56,330
48	Biopsy (tissue:foot)	Bone and joint	Positive	*Staphylococcus aureus*	Positive	*Staphylococcus aureus*	33 (18.4)	Low	Yes (1,239/8.4)	*Cutibacterium acnes (90/50.3), Moraxella osloensis (33/18.4), Staphylococcus hominis (8/4.5), Ralstonia solanacearum (6/3.4), Propionibacterium sp (4/2.2), Escherichia coli (4/2.2), Cutibacterium granulosum (3/1.7)*	34,560,878	22,847,071	179	25,677,418	17,132,707	14,801
49	Joint fluid	Bone and joint	Positive	*Staphylococcus aureus*	Positive	*Staphylococcus aureus*	36 (56.3)	Low	Yes (299/3.6)	*Cutibacterium acnes (18/28.1)*	30,830,034	20,161,727	64	25,634,338	15,724,781	8,266
50	Biopsy (tissue: spine)	Bone and joint	Positive	*Staphylococcus aureus*	Positive	*Staphylococcus aureus*	170,822 (99.9)	Intermediate	Yes (154,940/96.5)	X	34,869,438	23,106,922	170,850	1,588,880	722,767	160,520
51	Biopsy (tissue: valve prosthesis)	Cardiovascular	Positive	*Streptococcus milleri* group	Positive	*Streptococcus anginosus*	156 (75.7)	Low	Yes (96/0.8)	*Staphylococcus aureus (14/6.8), Ralstonia solanacearum (14/6.8), Klebsiella pneumoniae (4/1.9), Pseudomonas sp (4/1.9), Uncultured bacterium (3/1.5), Melaminivora sp (3/1.5)*	45,492,668	26,894,710	206	37,054,226	21,186,858	12,272
52	Biopsy (tissue: aortic valve)	Cardiovascular	Positive	*Streptococcus mitis* group	Positive	*Streptococcus sanguinis*	379 (66.5)	Intermediate	Yes (95/0.05)	*Cutibacterium acnes (38/6.7), Pseudomonas putida (14/2.5), Escherichia coli (12/2.1), Pseudomonas psychrotolerans (11/2.0), Acidovorax sp (11/2.0), Ralstonia solanacearum (10/1.75), Pseudomonas aeruginosa group (1.2), Thermus scotoductus (6/1.1)*	11,495,818	5,266,919	570	30,777,850	15,532,463	175,487
53	Biopsy (bone)	Bone and joint	Negative	X	Negative	X	X	X	X	*Ralstonia solanacearum (4/30.8), Staphylococcus aureus (3/23.1)*	24,627,924	14,908,581	13	26,745,638	16,570,264	29,479
54	Biopsy (tissue: ankle)	Bone and joint	Negative	X	Negative	X	X	X	X	*Kocuria rhizophila (7/16.7), Acinetobacter schindleri (9.5), Diaphorobacter sp (4/9.5)*	29,811,842	19,139,186	42	24,801,086	12,403,511	161,803
55	Joint fluid	Bone and joint	Negative	X	Negative	X	X	X	X	*Cutibacterium acnes (4/40.0)*	18,785,234	11,477,857	10	13,894,018	8,320,238	78,252

56	Joint fluid	Bone and joint	Negative	X	Negative	X	X	X	X	*Ralstonia solanacearum (4/28.6)*	30,321,000	18,910,315	14	16,642,658	6,108,776	22,239
57	Abscess (kidney)	Genito-urinary	Negative	X	Negative	X	X	X	X	*Acinetobacter junii (16/8.9), Pseudomonas aeruginosa group (14/7.8), Cutibacterium acnes (12/6.7), Ralstonia solanacearum (11/6.1), Achromobacter xylosoxidans (10/5.6), Escherichia coli (8/4.4), Rhodocyclaceae bacterium (7/3.9), Propionibacterium sp (6/3.3), Acetoanaerobium sticklandii (6/3.3), Haemophilus parainfluenzae (5/2.8), Alicycliphilus denitrificans (5/2.8), Acidovorax ebreus (5/2.8), Melaminivora sp (4/2.2), Bifidobacterium breve (4/2.2), Burkholderia cepacia complex (3/1.7), Acidovorax sp (3/1.7), Actinomyces oris (3/1.7)*	2,778,964	1,164,699	180	28,881,414	16,929,514	116,375
58	Biopsy (tissue: vegetation)	Cardiovascular	Positive	*Bartonella* sp.	Positive	*Bartonella quintana*	5,233,510 (99.9)	High	Yes (5,147,047/99.5)	X	12,036,808	2,066,350	5,233,529	30,139,064	3,002,421	5,175,257
59	Joint fluid	Bone and joint	Uninterpretable	X	Negative	X	X	X	X	*Ralstonia solanacearum (6/19.4), Citrobacter freundii complex (4/12.9), Arthrobacter sp (3/9.7)*	23,799,842	14,318,827	31	23,268,806	9,689,153	11,390
60	Biopsy (tissue: hip)	Bone and joint	Positive	*Escherichia coli*	Positive	*Escherichia coli*	337 (68.1)	Intermediate	Yes (160/6.1)	*Moraxella osloensis (36/7.3), Microbacterium aurum (26/5.3), Ralstonia solanacearum (12/2.4), Corynebacterium vitaeruminis (10/2.0)*	26,117,318	18,074,619	495	31,835,322	19,914,043	2,615
61	Abscess (intra-abdominal)	Intra-abdominal	Positive	*Staphylococcus* sp.	Positive	*Staphylococcus lugdunensis*	1,273 (79.7)	Intermediate	No	*Cutibacterium acnes (82/5.1), Escherichia coli (26/1.6), Pseudomonas sp (25/1.6), Ralstonia solanacearum (17/1.1)*	38,797,462	21,876,147	1,597	1,002,840	463,382	12
62	Biopsy (tissue: hip)	Bone and joint	Negative	X	Positive	*Cutibacterium acnes*	763 (99.6)	Intermediate	Yes (286/1.2)	*X*	20,851,744	15,058,863	766	15,451,286	10,571,474	24,584
63	Abscess (liver)	Intra-abdominal	Positive	*Fusobacterium nucleatum*	Positive	*Fusobacterium nucleatum*	2,594 (99.5)	Intermediate	Yes (12,400/96.0)	*X*	17,463,924	11,095,065	2,606	1,860,896	1,166,727	12,916
64	Biopsy (tissue: aortic valve)	Cardiovascular	Negative	X	Positive	*Staphylococcus epidermidis*	169 (44.4)	Low	Yes (1,698/28.7)	*Ralstonia solanacearum (48/12.6), Staphylococcus aureus (29/7.6), Cutibacterium acnes (18/4.7), Haemophilus parainfluenzae (10/2.6), Streptococcus sp (8/2.1), Streptococcus pneumoniae (6/1.6), Uncultured bacterium (6/1.6)*	74,903,930	49,731,363	381	32,084,336	13,359,404	59,252
65	Joint fluid (knee)	Bone and joint	Positive	*Streptococcus* sp. + *Moraxella* sp.	Negative	X	X	X	X	*Ralstonia solanacearum (10/35.7), Uncultured bacterium (8/28.6), Staphylococcus aureus (5/17.9)*	29,159,912	17,777,989	28	4,362,910	2,774,201	641
66	Abscess (knee)	Bone and joint	Positive	*Pseudomonas fluorescens* group	Negative	X	X	X	X	*X*	20,127,602	13,088,565	7	12,816	7,016	2
67	Biopsy (tissue: knee)	Bone and joint	Negative	X	Negative	X	X	X	X	*Ralstonia solanacearum (52/43.0), Staphylococcus aureus (32/26.4), Cutibacterium acnes (9/7.4), Escherichia coli (6/5.0), Microbacterium aurum (4/3.3)*	51,333,442	30,645,045	121	33,900,372	19,624,723	7,649

SMg: Shotgun metagenomics; Sanger 16S: Amplification of the 16S rRNA gene and Sanger sequencing of the PCR products
